# HIPAA Versus CIPA (California Invasion of Privacy Act): Are Physicians Protected from Live Social Media Streaming in the Emergency Department?

**DOI:** 10.5811/westjem.2020.2.47022

**Published:** 2020-04-13

**Authors:** Nicolas T. Sawyer, Maria Nguyen

**Affiliations:** * University of California, Davis, Department of Emergency Medicine, Davis, California; † University of California, Irvine, Department of Emergency Medicine, Orange, California

## The Problem

In a 2015 study by Elwin et al, approximately one in six patients stated they had secretly recorded a clinical interaction with their physician. Some patients stated they did it in hopes of replaying or relistening to the recording, while others stated they recorded their encounter to provide proof of their perceived negative healthcare experience. In the current age of ubiquitous Internet connectivity and the ability to share anything on social media at a moment’s notice, it is critical that physicians be aware of laws enacted to protect our safety and integrity as practicing clinicians in the 21^st^ century.^1^

What would *you* do if you discovered a patient had broadcast your clinical encounter in the emergency department (ED) live on social media? Who would you look to for help, and would they feel compelled to help you? What does the law say? In October 2018 this happened to me, and the answers to these questions may surprise you.

## The Scenario

Our team had briefly heard about the patient during sign out – she was a woman in her 30s who had been brought to the ED by police for agitation and public intoxication. Recent methamphetamine use exacerbated her untreated bipolar disorder, and our emergency psychiatric services team subsequently placed her on an involuntary 5150 hold for grave disability. During her medical clearance evaluation, she was incidentally found to be pregnant. From an obstetrics standpoint, she was asymptomatic and her medical workup was otherwise negative. She had been deemed medically cleared for transfer to an acute psychiatric hospital (APH) for inpatient mental health treatment and stabilization. Unfortunately, when the time came for transfer to the psychiatric hospital the patient refused transport and, thus, a member of our nursing staff asked if I would speak with her.

I walked over to the treatment area where the patient was being held. Originally intended for the evaluation and treatment of patients seeking emergency care, it had been converted into a boarding area used almost exclusively for holding psychiatric patients awaiting transport to an APH. All the room’s walls are stripped bare of the usual supplies, examining instruments, and monitors to prevent patients from attempting to hurt themselves or others. At times, unfunded patients and patients on Medi-Cal have remained in our ED awaiting placement for over 1000 hours due to APHs holding beds for private payers. Not only does this create a two-tiered system of psychiatric care, it is in violation of the EMTALA statute – a situation we have previously described as the “EMTALA loophole” in psychiatric care.^2^

Nothing appeared particularly out of the ordinary as I entered the patient’s room. A disheveled female of approximate stated age sat on the gurney with her legs crossed wearing a hospital gown, and a blanket was crumpled between her legs. She was clearly upset, ranting loudly, which I presumed was her responding to internal stimuli. “Hello Ms. Smith, I’m Dr. Sawyer, it is nice meeting you,” I said as I entered the room. “You too. Excuse me I’m exposed,” she said as she proceeded to readjust the blanket between her legs. Little did I know that the patient’s shouting I had heard as I entered the room was not her responding to internal stimuli or mere agitation, but rather she was speaking into her cell phone that was hidden in the blanket between her legs with the camera looking upward at her face. She was broadcasting her frustrations on Facebook Live.

As I sat down to speak with the patient, I began by saying that I had been told she had refused transport. She responded, “I certainly do. I disagree with everything, there was no reason for this, it was totally uncalled for. I was just on my phone a second ago and I was trying to get help from my council member. All you did was cause a bill that was uncalled for.” I explained that our emergency psychiatric services team who evaluated her was concerned enough to place her on an involuntary hold. I explained that the best way to resolve the issue was to allow for transfer and evaluation by the specialists at the APH. She again refused. Ultimately, we reached an impasse, and I explained that I would get the psychiatric team to come speak with us to help get the issue resolved. But before I left the room, she revealed the phone she had been hiding in the sheets between her legs—battery now dead—and told me she had broadcast our entire conversation on Facebook Live.

She wasn’t bluffing. After leaving the patient’s room I searched her name on Facebook and found the video of our conversation on her public page. Similar to other social media platforms that support live streaming, including Twitter’s Periscope, YouTube Live, and Instagram’s live video streaming option, not only are the user’s followers notified when the user “goes live,” but after the live broadcast concludes, the recording remains on the user’s page in perpetuity unless the user chooses to delete it. At that time, I made a screen recording of her Facebook Live post, which allowed me to transcribe her words for this article verbatim.

I wasn’t concerned about my interaction with the patient. Even before reviewing the video, I was confident that I had conducted myself in a professional manner. However, secretly broadcasting this otherwise-private conversation without my knowledge or consent was highly concerning for two reasons: 1) she was coherent enough to make allegations that, when taken out of context, could be interpreted as physician mistreatment of a vulnerable patient; and 2) I knew I had introduced myself by name—as I always do—and this could focus any potential public backlash directly on me. This potential scenario was confirmed almost instantly—she had over 500 followers, her post was open to the public and shareable, and within one hour of publication had 52 views and nine comments. The first eight comments focused on the patient’s well being, but the ninth comment was filled with expletives and criticism aimed at me and the hospital where I practice.

## The Consequences and Conundrum

I returned to the patient’s room and asked her if she would delete her post. She stated that her phone’s battery was now dead and even if it were not, she would not delete it. She appeared satisfied in her decision to broadcast her conversation, as if she had won some twisted new game she had created and used to ensnare me. I had no idea what to do. I called in the police.

In the interim, I managed to get the patient’s phone from her by explaining that I would charge it and return it to her. I was hoping the phone would not be password protected (an admittedly insanely low probability), and I could enter her Facebook app and delete the post myself. At that time, emergency medical services arrived to transfer the patient to the APH. Unsurprisingly, the patient was no longer refusing transport but upon multiple requests continued to refuse my requests to delete her Facebook post. I asked them not to leave as the two police officers had just arrived and I wanted to speak with them first.

What ensued was a complicated series of interactions with our hospital’s dedicated police officers, our nursing supervisor, our department’s medical director, and ultimately risk management. Our attorney informed me that the risk management department would contact Facebook the following morning to request the video be taken offline, but there was no guarantee that Facebook would take any action. While I appreciated the assistance of all involved, as well as the difficulty of navigating this novel situation without guidance from standing policies and procedures, the recurring message I received was that all existing policies tended to favor patient confidentiality as mandated by the Health Insurance Portability and Accountability Act of 1996 (HIPAA). With the exception of our department’s medical director, I felt there was very little, if any, consideration regarding the prospect of endangering the physician involved. But what does the law say?

## The Law on this Situation

According to an article published in 2017 by Elwyn et al in the *Journal of the American Medical Association* entitled, “Can patients make recordings of medical encounters? What does the law say?,” state wiretapping or eavesdropping laws provide guidance as to whether a patient may record his or her interaction with a medical provider without the provider’s consent.^3^ In Texas, Oregon, and 37 other states, the consent of just one party in the interaction is sufficient for the recording and its distribution to be lawful (Figure). Therefore, a patient in one of these “one-party” states has the right to record a clinical encounter without the healthcare provider’s consent, and without the likelihood of legal sanction.

However, within the remaining 11 states including California and Washington—also known as “all-party jurisdiction states”—state law dictates that *all* parties recorded must express their consent. This thereby makes covert recordings illegal. The California Invasion of Privacy Act (CIPA), was enacted in 1967 “to protect the right of privacy of the people of this state.” Noting the advent of new devices and technology used “for the purposes of eavesdropping upon private communications,” the California State Legislature stated that the “use of such devices and techniques has created a serious threat to the free exercise of personal liabilities and cannot be tolerated in a free and civilized society.”^4^ CIPA was updated in 2016 with the passage of Assembly Bill (AB) 1671 in response to the covert recording of Planned Parenthood providers used to create a false narrative about the organization.^5^ AB 1671, which became effective on January 1, 2017, states that any person who “discloses or distributes, in any manner...including, but not limited to, internet web sites and social media...the contents of a confidential communication with a health care provider...be punished by a fine not exceeding $2,500 per violation, or imprisonment in a county jail not exceeding one year.”^6^

Unfortunately, no one I spoke with that evening was aware of the protections afforded to practitioners under AB 1671 nor had any of us encountered this situation before. While I believe that everyone involved did their best to help resolve this unique dilemma, I was uncomfortable with the idea that the video would remain on Facebook overnight and potentially forever. So, in a last-ditch effort to resolve the issue I returned to the patient’s room, sat down, apologized for any misunderstandings and asked the patient if she would please delete the recording. After about 15 minutes of intensive active listening and engagement, I was able to earn her trust and she allowed me to take her through the steps required to delete the video from her Facebook account. I felt tremendously relieved, but I also wanted to ensure that this didn’t happen to my colleagues or if it did, that they would be aware of their rights.

## The Aftermath: Development of Institutional Policy

Since then, I have worked with our hospital’s leadership to ensure not only that our governing policies reflect the privacy laws protecting our state’s healthcare providers, but also that our employees are made aware of these laws. We have neared completion of a revised institutional authorization and consent to photograph or interview policy to now outline in a step-by-step manner the actions an employee should take if he or she is ever recorded without their consent. It now also acknowledges live broadcasting on social media platforms as a form of recording, which, like any digital image or recording device, is prohibited and subject to legal sanctions without the all party’s consent. Furthermore, we have summarized these policies in a digital flier that outlines our employees’ privacy rights, the concerns to be aware of should they agree to be recorded, and the actions to take if they learn that they have been recorded. In the near future, our policy will serve to protect not only our patients, but those who work tirelessly to care for them.

## Figures and Tables

**Figure:**
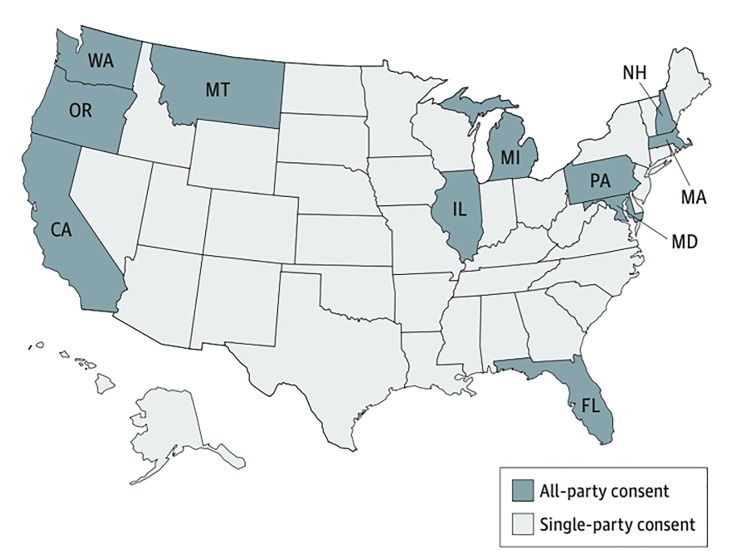
US States requiring all-party or single-party consent for audio recording of conversations.^3^ Figure obtained from: Elwyn G, Barr PJ, Castaldo M. Can patients make recordings of medical encounters? What does the law say? *JAMA*. 2017;318(6):513–14.
